# Apicidin attenuates memory deficits by reducing the Aβ load in APP/PS1 mice

**DOI:** 10.1111/cns.14102

**Published:** 2023-01-27

**Authors:** Biao Luo, Jian Chen, Gui‐Feng Zhou, Xiao‐Yong Xie, Jing Tang, Qi‐Xin Wen, Li Song, Shi‐Qi Xie, Yan Long, Guo‐Jun Chen, Xiao‐Tong Hu

**Affiliations:** ^1^ Department of Neurology The First Affiliated Hospital of Chongqing Medical University, Chongqing Key Laboratory of Neurology Chongqing China; ^2^ Department of Health Management, Daping Hospital Army Medical University Chongqing China; ^3^ Department of Neurology The Ninth People's Hospital of Chongqing Chongqing China

**Keywords:** ADAM10, Alzheimer's disease, apicidin, histone deacetylase inhibitor, phosphorylation tau

## Abstract

**Aims:**

Amyloid beta (Aβ) is an important pathological feature of Alzheimer's disease (AD). A disintegrin and metalloproteinase 10 (ADAM10) can reduce the production of toxic Aβ by activating the nonamyloidogenic pathway of amyloid precursor protein (APP). We previously found that apicidin, which is a histone deacetylase (HDAC) inhibitor, can promote the expression of ADAM10 and reduce the production of Aβ in vitro. This study was designed to determine the potential of apicidin treatment to reverse learning and memory impairments in an AD mouse model and the possible correlation of these effects with ADAM10.

**Methods:**

Nine‐month‐old APP/PS1 mice and C57 mice received intraperitoneal injections of apicidin or vehicle for 2 months. At 11 months of age, we evaluated the memory performance of mice with Morris water maze (MWM) and context fear conditioning tests. The Aβ levels were assessed in mouse brain using the immunohistochemical method and ELISA. The expression of corresponding protein involved in proteolytic processing of APP and the phosphorylation of tau were assessed by Western blotting.

**Results:**

Apicidin reversed the deficits of spatial reference memory and contextual fear memory, attenuated the formation of Aβ‐enriched plaques, and decreased the levels of soluble and insoluble Aβ40/42 in APP/PS1 mice. Moreover, apicidin significantly increased the expression of ADAM10, improved the level of sAPPα, and reduced the production of sAPPβ, but did not affect the levels of phosphorylated tau in APP/PS1 mice.

**Conclusion:**

Apicidin significantly improves the AD symptoms of APP/PS1 mice by regulating the expression of ADAM10, which may contribute to decreasing the levels of Aβ rather than decreasing the phosphorylation of tau.

## Graphical abstract

Apicidin improves cognitive deficits in APP/PS1 mice by promoting ADAM10 expression. The α‐site cleavage of APP by ADAM10 generates more sAPPα relative to sAPPβ, leading to the decreased Aβ burden, which contributes to the reversal of memory deficits in AD. The present work highlights the crucial role of histone deacetylase inhibitors in alleviating AD‐like pathology and supports that ADAM10 could serve as a disease‐modifying and therapeutic target.

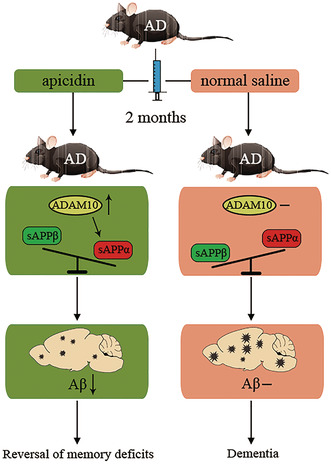

## 1 INTRODUCTION

Alzheimer's disease (AD) is a type of dementia that is associated with the deterioration of brain functions, and AD accounts for 50%–60% of dementia cases. Currently, over 46 million people are living with dementia worldwide. This number is estimated to increase to 131.5 million by 2050.^1^ The primary pathological characteristics of AD include senile plaques (SPs), which are caused by the self‐assembly of amyloid‐β (Aβ) peptides, and neurofibrillary tangles (NFTs), which arise due to abnormally phosphorylated tau proteins; these factors contribute to cognitive decline and memory loss.^2^, ^3^, ^4^ Moreover, increasing evidence suggests a key role for dyshomeostasis between the production and clearance of Aβ during AD‐related pathological alterations; this phenomenon is an important early, and often initiating, factor of disease.^5^ Aβ is a metabolite of the successive cleavage of amyloid precursor protein (APP) by β‐secretase (BACE1) and γ‐secretase, and this process, which is called the amyloid metabolic pathway, contributes to the pathology of AD. In contrast, α‐secretase (ADAM10), which is a component of the nonamyloidogenic pathway, cleaves a region of APP that prevents Aβ formation and aggregation in the brain.^6^ Based on the effects of multiple secretases on Aβ, extensive studies have focused on developing secretase modulators for use in the treatment of AD; some of these modulators have shown success in clinical trials, while others have failed.^7^

Both genetic and nongenetic factors contribute to the etiopathogenesis of AD. Nongenetic mechanisms refer to DNA modifications that do not alter the base DNA sequence of AD‐related genes; these modifications are also called epigenetic modifications. Recently, epigenetic modification in AD has become a hot topic in studies related to the pathophysiology of AD and novel therapies.^8^, ^9^ Among the common epigenetic mechanisms, histone modification, via phosphorylation, acetylation, ubiquitylation, and sumoylation, is one of the most important types of epigenetic modifications. In comparison, acetylation/deacetylation of histones is the most well‐studied histone modification in AD therapy. Extensive experimental evidence indicates decreased histone acetylation levels in various neurodegenerative disorders.^10^, ^11^ Presently, multiple histone deacetylase (HDAC) inhibitors have shown the ability to reverse the pathological hallmarks of AD and have produced good results in vitro and in vivo.^12^, ^13^, ^14^, ^15^

Apicidin, as an HDAC inhibitor, selectively binds to class I HDACs and interferes with the deacetylation process.^16^ Apicidin has been found to inhibit parasites and tumor proliferation and to activate innate immune effectors.^17^, ^18^, ^19^ In a previous study, we found that apicidin increased the expression of ADAM10 in cultured cells. This effect was mediated by the transcription factor USF1 and was dependent on the ERK signaling pathway.^20^ Several studies have shown that ADAM10 protein expression is decreased in the CSF of AD patients.^21^, ^22^ Moreover, the proteolytic processing of APP by ADAM10 produces a secreted ectodomain fragment (sAPPα) that has neuroprotective and neurotrophic properties.^23^ Stimulation of ADAM10 activity or ADAM10 overexpression is a suitable therapeutic approach for AD.^24^, ^25^ Considering that apicidin can improve histone acetylation levels and increase the expression of ADAM10, it could be an effective treatment for AD. Therefore, we investigated the potential of apicidin treatment to reverse learning and memory impairments in an AD mouse model and the possible correlation of these effects with ADAM10.

## 2 MATERIALS AND METHODS

### 2.1 Animal treatment and sample preparation

All protocols were approved by the Commission of Chongqing Medical University for the ethics of experiments on animals and were conducted in accordance with international standards. The amyloid precursor protein (APPswe)/presenilin 1 (PSEN1dE9) heterozygous transgenic mice (APP/PS1, B6C3) and wild‐type mice (C57BL/6; C3H) were obtained from Jackson Laboratory imported by Nanjing University. The animals were housed in a temperature‐controlled animal facility with a 12 h light–dark cycle (lights on at 6:00 a.m.). Water and food were freely available in their home cages. All procedures followed the National Institutes of Health (NIH) Guide for the Care and Use of Laboratory Animals (NIH Publications No. 80‐23, revised 1996) and were approved by the Animal Care and Use Committee of Ningbo University (Ningbo, China). All efforts were made to minimize animal suffering.

To collect brain samples, mice were euthanized with pentobarbital and then transcardially perfused with ice‐cold phosphate‐buffered saline (PBS). A total of 28 male mice were randomly assigned into four groups (*n* = 7 per group): wild‐type (WT) and APP/PS1 (AD) mice that were treated with normal saline (ns) or apicidin (ap) for 2 months, respectively. A whole brain was then separated into two hemispheres, leading to 14 samples in each group. Whereas four hemispheres were fixed by 4% paraformaldehyde (PFA) for immunohistochemistry study, the hippocampus and cortex from remaining hemispheres were rapidly isolated and stored in liquid nitrogen for either ELISA (*n* = 4) or Western blotting (*n* = 6) analysis.

### 2.2 Drugs and treatment

**FIGURE 1:**
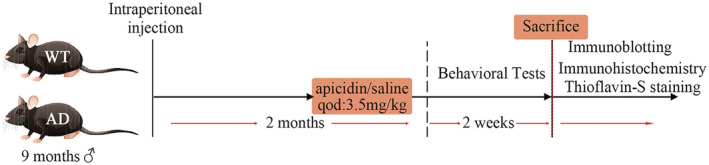
Schematic illustrations show the overall timeline and experimental design.

Apicidin {cyclo[((2S)‐2‐amino‐8‐oxodecanoyl)‐N1‐methoxy‐L‐tryptophyl‐L‐isoleucyl‐D‐homoprolyl]}^15^ with 98% HPLC purity was obtained from GLPBIO (Montclair, California, USA). Apicidin was dissolved in 5% Tween 80 to generate a 20 mM stock solution and stored at −80°C. Subsequent dilutions were made in saline (0.9%). Apicidin was given an intraperitoneal injection every 2 days for 2 month, and control groups received an equal volume of vehicles. The schedule of drug treatment and test orders is shown in Figure 1A.

### 2.3 Morris water maze

The effect of apicidin treatment on spatial learning and memory was assessed by MWM testing at the age of 11 month. A stainless pool with a diameter of 100 cm and a height of 50 cm containing a submerged escape platform (10 cm in diameter) 1.5 cm below the water surface was used. The water temperature was kept at 23 ± 1°C and made opaque by the addition of non‐toxic white titanium dioxide. The pool was arbitrarily divided into four quadrants (training, adjacent left, adjacent right, and opposite). The mice were first introduced to the visible platform on day one to habituate them to the experimental environment and trained for four trials in which the platform location and starting direction differ with each trial. The platform was then moved to another quadrant and hidden beneath the surface of the water. Mice were then given four trials per day for seven consecutive days to measure their ability to navigate to the hidden platform, beginning from separate quadrants with their heads facing the wall. Each trial ended either when an animal climbed onto the platform or when a maximum of 60 s had elapsed. Next, they were allowed to remain on the platform for 10 s. Twenty‐four hours after the last training session, all mice were subjected to a probe trial in which they swam for 60 s in the pool with no platform, and the time taken to cross to the quadrant originally containing the platform was assessed. Mice were monitored by a camera mounted on the ceiling directly above the pool, and all trials were recorded using a water maze program for subsequent analysis of escape latencies, swimming speed, path length, and percent time spent in each quadrant of the pool during probe trials (analysis program).

### 2.4 Fear conditioning

Mice were tested in a 3‐day paradigm. On the first day, the mice were placed in the training chamber to acclimate for 120 s, and then they were presented with a 30 s pure tone (4500 Hz, 60 dB) that co‐terminated with 2 s foot shock (0.45 mA). This procedure was repeated three times with an intertrial interval of 120 s. On the second day, the mice were returned to the training chamber for 10 min without exposure to either the tone or foot shock to measure contextual freezing. On the last day, the mice were placed in the training chamber for 120 s, then presented with a 30 s pure tone (4500 Hz) without the 2 s foot shock. Behavior was recorded by video camera, and freezing data were measured using SuperFcs software.

### 2.5 Immunohistochemistry

Briefly, hemibrains were cut into paraffin sections (4 μm), followed by dewaxing and rehydration. Then, sections were boiled for 15 min in citrate buffer (10 mM) at pH 6.0 by heating in a microwave and cooled naturally. Next, they were incubated with H_2_O_2_ (3% in PBS) for 10 min to inhibit endogenous peroxidases and blocked for 1 h with blocking buffer (5% BSA) at room temperature, followed by incubation with a mouse monoclonal anti‐β‐Amyloid 6E10 (1:1000; SIG‐39300, Covance) overnight at 4°C. On the next day, sections were incubated with a secondary antibody (HRP‐conjugated goat anti‐mouse IgG, Protech) for 1 h at 37°C and finally reacted with DAB (Zhongshan Golden Bridge) and stained by hematoxylin. Images were captured using a light microscope (IX71, Olympus). For the hippocampal or cortical region, a photo of the entire hippocampus or cortex of each brain was captured, and the number and area of Aβ plaques were counted. Four hemibrains were involved in each group. The number and area of Aβ plaques in the cortex and hippocampus were counted in a blinded manner using Image‐Pro Plus software.

### 2.6 Thioflavin S staining

Hemibrain tissue paraffin sections (as described previously) were allowed to treat with the clearing agent xylene (paraffin solvent) and a series of graded EtOH for deparaffinization and hydration. Later, the brain sections were incubated with 0.3% thioflavin S (Sigma‐Aldrich) for 20 min at room temperature in the dark. Subsequently, these were submitted to washes in 3 min series, specifically with 80% ethanol (two washes), 90% ethanol (one wash), and three washes with PBS. Finally, the slides were mounted using Fluoromount (EMS), allowed to dry overnight at room temperature in the dark, and stored at 4°C. Image acquisition was performed with an epifluorescence microscope (Olympus, Germany). Plaque quantification in the cortex and hippocampus were counted in a blinded manner using Image‐Pro Plus software.

### 2.7 Western blotting

Brain tissues were homogenized and sonicated in ice‐cold lysis buffer [50 mM Tris–HCl, pH 8.0, 140 mM NaCl; 1.5 mM MgCl_2_; 0.5% NP‐40 with complete protease inhibitor cocktail (Roche)]. Protein concentration was measured in the supernatant by BCA Protein Assay (Dingguo). The monoclonal or polyclonal antibodies used were BACE1 (Abcam, 1:1000); APP full length (A8717, Sigma, 1:3000); sAPPα (6E10, Covance, 1:1000); sAPPβ (Covance, 1:500); ADAM10 (Abcam, 1:1000); PSEN1 (Proteintech, 1:500); GAPDH (Proteintech, 1:10000); tau46 (CST, 1:1000); phosphorylation‐tau 181 (CST, 1:1000); phosphorylation‐tau 202 (Abcam, 1:5000); and phosphorylation‐tau 396 (Abcam, 1:10000). The secondary antibodies were goat anti‐rabbit or anti‐mouse horseradish peroxidase‐labeled antibodies (Proteintech, 1:5000). The membranes were visualized using an ECL reagent (Thermo) and a Fusion FX5 image analysis system (VilberLourmat). Relative protein intensities were calculated using Quantity One software (Bio‐Rad).

### 2.8 ELISA‐based measurement of Aβ level

Brain tissues were homogenized in PBS with protease inhibitor. Soluble Aβ was present in the supernatant after homogenization and 10 min centrifugation at 20,000 *g*. Insoluble Aβ, mostly in amyloid plaques, was incubated with 20 μL of 70% formic acid, mechanically dissociated with a micropipette, gently agitated for 1 h, and buffered with 380 μL of 1 M Tris–HCl (pH 8.0). Samples were centrifuged for 90 min at 20,000 *g*, and supernatants were collected for analysis.^26^ Soluble and insoluble amyloid‐β40 and amyloid‐β42 were measured by commercially available ELISA kits (Elabscience, Wuhan, China) according to the manufacturer's instructions. And total protein level was measured at 450 nm and recorded with an ultraviolet spectrophotometer.

### 2.9 Statistical analysis

All data were tested by Shapiro–Wilk or Kolmogorov–Smirnov normality test. Unpaired student's *t*‐test was used for comparisons of two means. Two‐way ANOVA with either Bonferroni's or Tukey's post hoc analysis was used for multiple comparisons when more than two means were being compared. Repeated‐measures three‐way ANOVA with Tukey's post hoc analysis was used to analyze changes in the mean of two independent variables. As a result, Bonferroni's or Tukey's adjusted *p* values are presented. For follow‐up, adjusted *p* < 0.05 was deemed to be of statistical significance. Data were graphed and analyzed using GraphPad Prism 9.0.

## 3 RESULTS

### 3.1 Apicidin treatment improved memory deficits in APP/PS1 mice

A cohort of 9‐month‐old APP/PS1 (AD) mice and wild‐type (WT) mice received intraperitoneal injections of vehicle (5% Tween 80 in 0.9% normal saline) or apicidin (3.5 mg/kg in 5% Tween 80 in 0.9% normal saline) every 2 days for two mo. At 11 month of age, we first evaluated the memory performance of mice with the MWM and context fear conditioning tests to determine whether apicidin can improve memory deficits. All the treatments continued during the behavioral testing period (Figure 1A).

**FIGURE 2:**
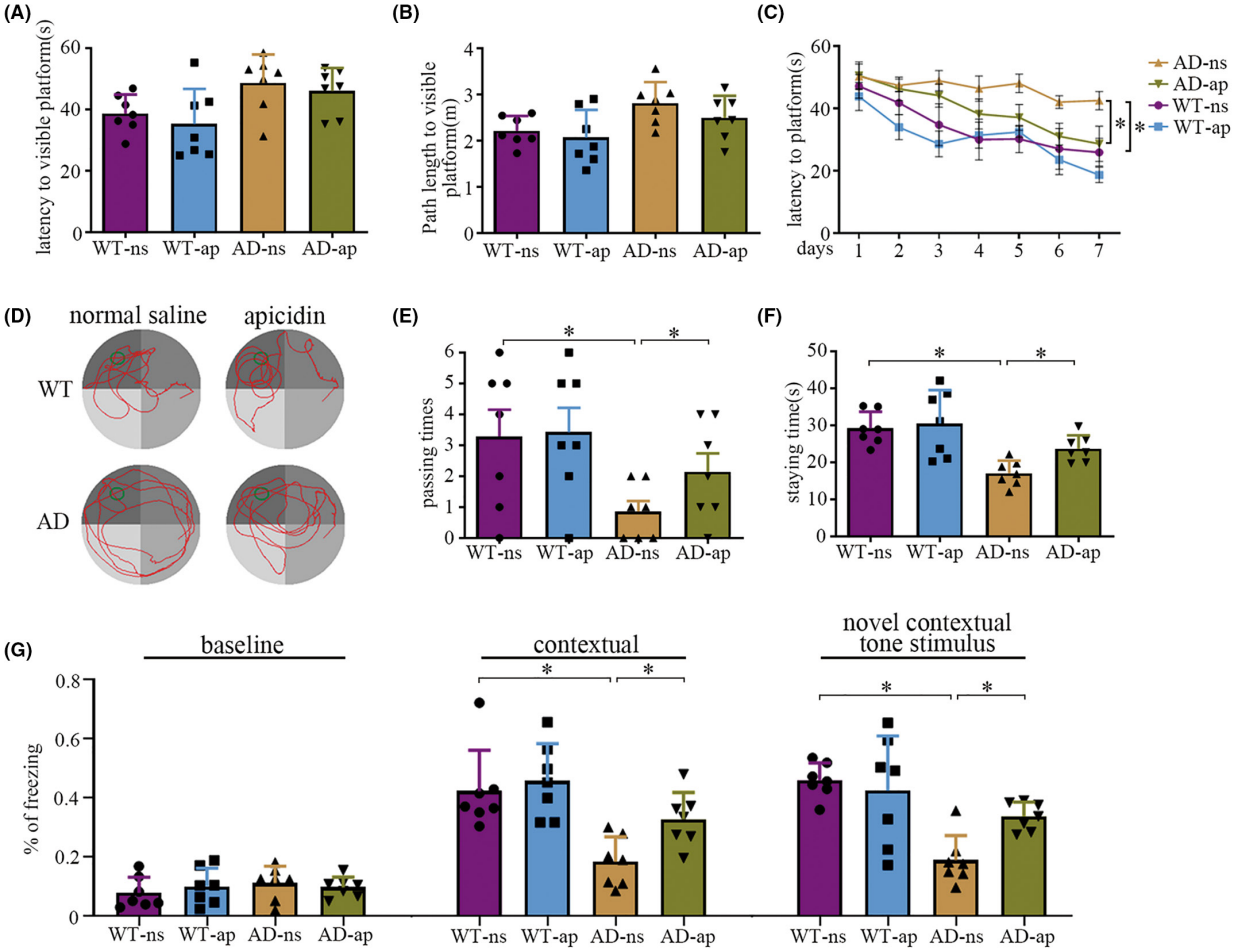
Apicidin improves spatial and contextual memory in APP/PS1 mice. (A) During visible platform tests, the apicidin (ap) and normal saline (ns) treated APP/PS1 (AD‐ns, AD‐ap) or wild‐type (WT‐ns, WT‐ap) mice exhibited a similar latency to escape onto the visible platform. (B) AD‐ns and AD‐ap mice or WT‐ns and WT‐ap mice had similar swimming distances before escaping onto the visible platform. (C) In the hidden platform tests, compared with AD‐ns mice, AD‐ap mice showed a significantly shorter latency on the fifth, sixth, and seventh days. Significant differences in latency were found for AD‐ns and WT‐ns mice. (D) Representative swimming trajectories in the probe trial. (E and F) In the probe trial, AD‐ap mice crossed significantly more times traveling (E) and spent a significantly longer time staying (F) in the place where the hidden platform was previously placed than AD‐ns mice. (G) In the context of fear conditioning tests, significant differences were found for AD mice and WT mice. Compared with AD‐ns mice, AD‐ap mice showed significant differences on the second day (contextual conditioning) and third day (tone stimulus). Two‐way ANOVA or three‐way repeat ANOVA with multiple comparisons test was used for all comparisons. The graphs represent mean ± SEM. *p < 0.05; *n* = 7.

To exclude the effects of the visual and motor abilities of the mice on learning and memory performance, the cued learning task was performed. In this task, a platform was made visible by a flag. Two‐way ANOVA revealed no significant effect of apicidin treatment on either latency [*F*(1, 24) = 0.8386, *p* = 0.3689] or path length [*F*(1, 24) = 1.682, *p* = 0.2070], indicating that apicidin did not affect the visual, motor, or motivational behaviors in WT mice or AD mice (Figure 2A,B). Nevertheless, there was a statistically significant difference between the WT mice and AD mice in both latency [*F*(1, 24) = 0.9687, *p* = 0.0047] and path length [*F*(1, 24) = 8.309, *p* = 0.0082] (Figure S1A,B). Next, hidden platform tests were initiated and continued throughout the course of 7 days with all the mice (Figure 2C). Three‐way repeated ANOVA revealed a significant difference in latency to find the hidden platform between different treatments [*F*(1, 24) = 4.881, *p* = 0.0369], between different genotypes [*F*(1, 24) = 20.72, *p* = 0.0001] and across different days [*F*(6, 144) = 11.67, *p* < 0.0001]. The interaction of the treatment, day, and genotype in affecting the latency did not differ significantly [*F*(1, 144) = 0.9657, *p* = 0.4507]. There were significant differences between the AD‐ns group and AD‐ap group and between the WT‐ns group and AD‐ns group according to Tukey's multiple‐comparisons test [AD‐ns vs AD‐ap, *p* = 0.0469, WT‐ns vs. AD‐ns, *p* = 0.0010] (Figure 2C). When the platform was removed during the probe trial, the AD mice treated with normal saline showed a turbulent trajectory compared with the WT mice. However, the AD mice treated with apicidin showed some improvement (Figure 2D). In addition, two‐way ANOVA revealed a significant difference in the effects of mouse genotype on the number of times [*F*(1, 24) = 7.539, *p* = 0.0113] the target platform was crossed and the time [*F*(1, 24) = 20.09, *p* = 0.0002] spent in the target quadrant (Figure 2E,F). Moreover, the number of times [*p* = 0.0205] the target platform was crossed and the time [*p* = 0.0358] spent in the target quadrant were significantly increased in the AD‐ap mice compared with the AD‐ns according to multiple‐comparisons analysis (Figure 2E,F). No significant differences were observed between the WT mice treated with apicidin or normal saline in terms of escape latency, number of times the target was crossed, or time spent in the target quadrant. The subsequent context fear conditioning tests revealed that the WT mice exhibited a longer freezing time than the AD mice in the contextual conditioning [*F*(1, 24) = 18.92, *p* = 0.0002] and tone stimulus [*F*(1, 24) = 19.07, *p* = 0.002] tests (Figure 2G). There were significant differences in the AD‐ns group compared to the AD‐ap group according to Tukey's multiple‐comparisons test [contextual conditioning, *p* = 0.0488; tone stimulus, *p* = 0.0341]. No significant differences were observed between the WT‐ap and WT‐ns mice in terms of the length of freezing times. These results indicated that apicidin significantly improved spatial and associative learning memory in AD mice.

### 3.2 Treatment with apicidin attenuated the formation of Aβ‐enriched plaques in the brains of APP/PS1 mice

**FIGURE 3:**
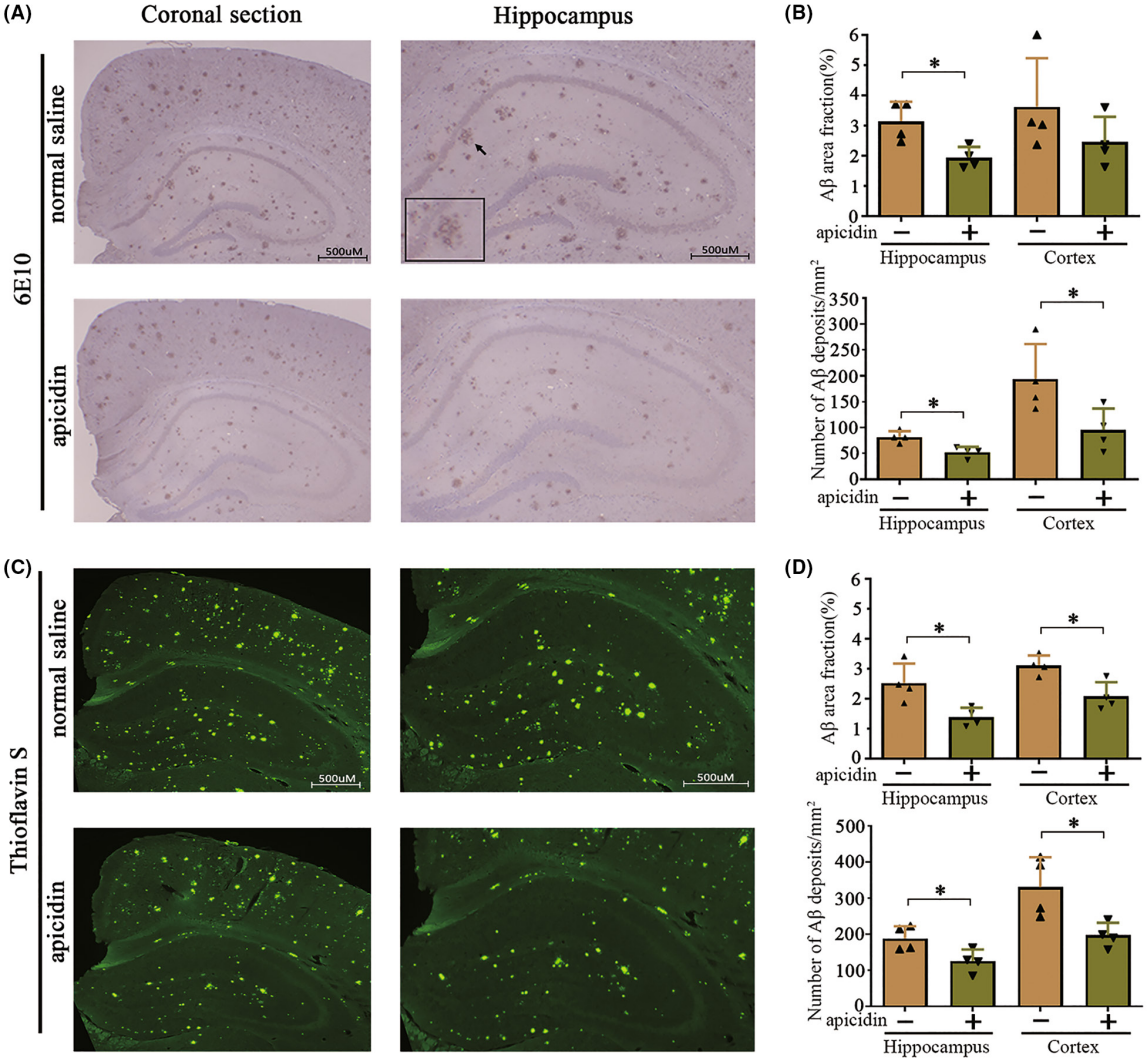
Apicidin treatment relieves Aβ plaques in APP/PS1 mice. (A) Representative immunohistochemical images of 6E10‐labeled Aβ burden in the coronal and hippocampal sections of AD‐ap and AD‐ns mice. (B) Quantification of the number (bottom) and percent area (top) of 6E10‐labeled Aβ plaques in the hippocampus and cortex. (C) Representative images of Thioflavin S staining positive compact plaques in the coronal and hippocampal sections of AD‐ap and AD‐ns mice. (D) Quantification of the number (bottom) and percent area (top) of Thioflavin S staining positive compact plaques in the hippocampus and cortex. Two‐way ANOVA with multiple comparisons test and unpaired student's *t*‐test was used for analysis. The graphs represent mean ± SEM. *p < 0.05; *n* = 4.

**FIGURE 4:**
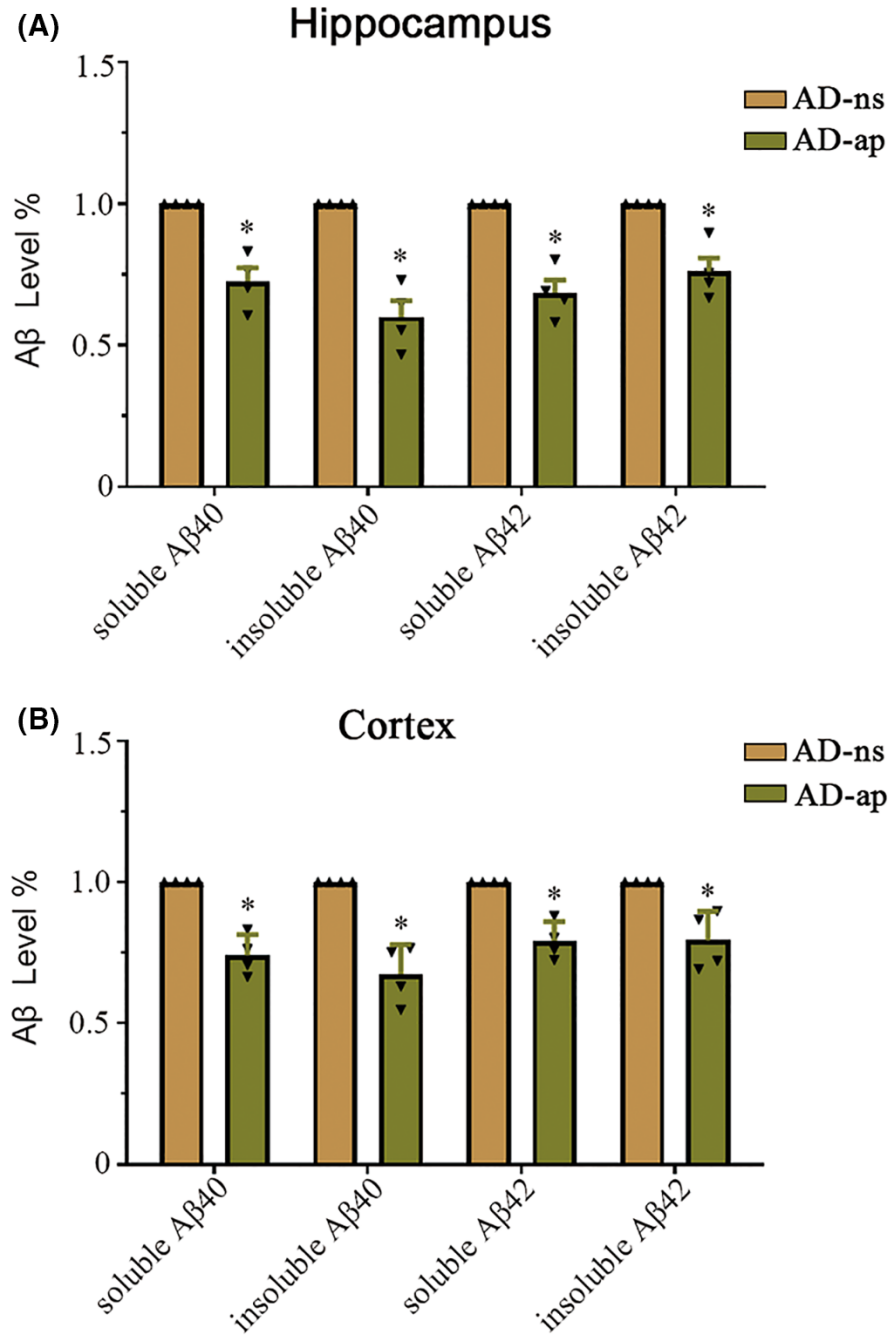
Amyloid‐β (Aβ) level in the brains of APP/PS1 mice. (A and B) ELISAs were used to measure soluble and insoluble amyloid‐β 40/42 levels in the hippocampus (A) and cortex (B) of AD‐ap and AD‐ns mice. Unpaired student's *t*‐test was used for all comparisons. The graphs represent mean ± SEM. *p < 0.05; *n* = 4.

The brains of AD patients and mouse models of AD are characterized by amyloid plaque accumulation followed by neuronal death. Aβ plaque deposition is the main pathological feature observed in the brains of AD mice. Next, we examined whether apicidin reverses amyloid plaque formation in AD mice. We assessed the number and percent area covered by Aβ plaques in the cortex and hippocampus of mice using a 6E10 antibody that recognizes all amyloid‐β species. The representative image in Figure 3A illustrates the effect of apicidin in the brains of mice using the immunohistochemical method. Two‐way ANOVA with multiple comparisons was conducted to compare the effect of apicidin on the maximum amount of Aβ and the largest percent area covered by Aβ, as shown in Figure 3B. First, abundant Aβ plaques were observed in the AD mice than in the WT mice (Figure S2A). Notably, apicidin treatment reduced the maximum number of Aβ plaques [*p* = 0.0012] and the largest percent Aβ area [*p* = 0.0003] covered by Aβ in the hippocampus (Figure 3B). Although the Aβ area showed a decreasing trend, no significant differences were observed in the cortices of AD mice in the AD‐ns and AD‐ap groups [*p* = 0.1819] (Figure 3B). Next, to obtain more reliable results, thioflavin S staining was used to detect Aβ aggregation and amyloid plaque formation. Consistent with previous results, thioflavin‐S staining revealed that Aβ aggregation and amyloid plaque formation were significantly reduced in the AD mice that received apicidin (Figure 3C), and the WT mice samples were not positive for thioflavin‐S staining (Figure S2B). The quantified result of the maximum number of Aβ plaques [Hippocampus: *p* = 0.0264, Cortex: *p* = 0.0252] and the largest percent area covered by Aβ [Hippocampus: *p* = 0.0202, Cortex: *p* = 0.0117] in Figure 3D, and the results were analyzed by Unpaired student's *t*‐test. Aβ40 and Aβ42 are two major C‐terminal variants of the Aβ protein constituting the majority of Aβ plaques. Therefore, we used ELISA quantitative analysis to measure the Aβ40/Aβ42 levels in the homogenates of the hippocampus and cortex. Unpaired student's t test revealed decreased levels of soluble and insoluble Aβ40/42 in the hippocampus [soluble Aβ40, *t*(6) = 5.725, *p* = 0.0012; insoluble Aβ40, *t*(6) = 7.013, *p* = 0.0004; soluble Aβ42, *t*(6) = 6.872, *p* = 0.0005; insoluble Aβ42, *t*(6) = 4.896, *p* = 0.0027] and cortex [soluble Aβ40, *t*(6) = 7.105, *p* = 0.0004; insoluble Aβ40, *t*(6) = 6.249, *p* = 0.0008; soluble Aβ42, *t*(6) = 6.155, *p* = 0.0008; insoluble Aβ42, *t*(6) = 4.008, *p* = 0.0071] of the AD mice that had been treated with apicidin compared with those of the normal saline treated control mice (Figure 4A,B). The ELISA results are consistent with the immunohistochemistry results, indicating that the Aβ burden in the brains of the APP/PS1 mice was inhibited by treatment with apicidin.

### 3.3 Apicidin alters the APP processing pathway in the brains of APP/PS1 mice

**FIGURE 5:**
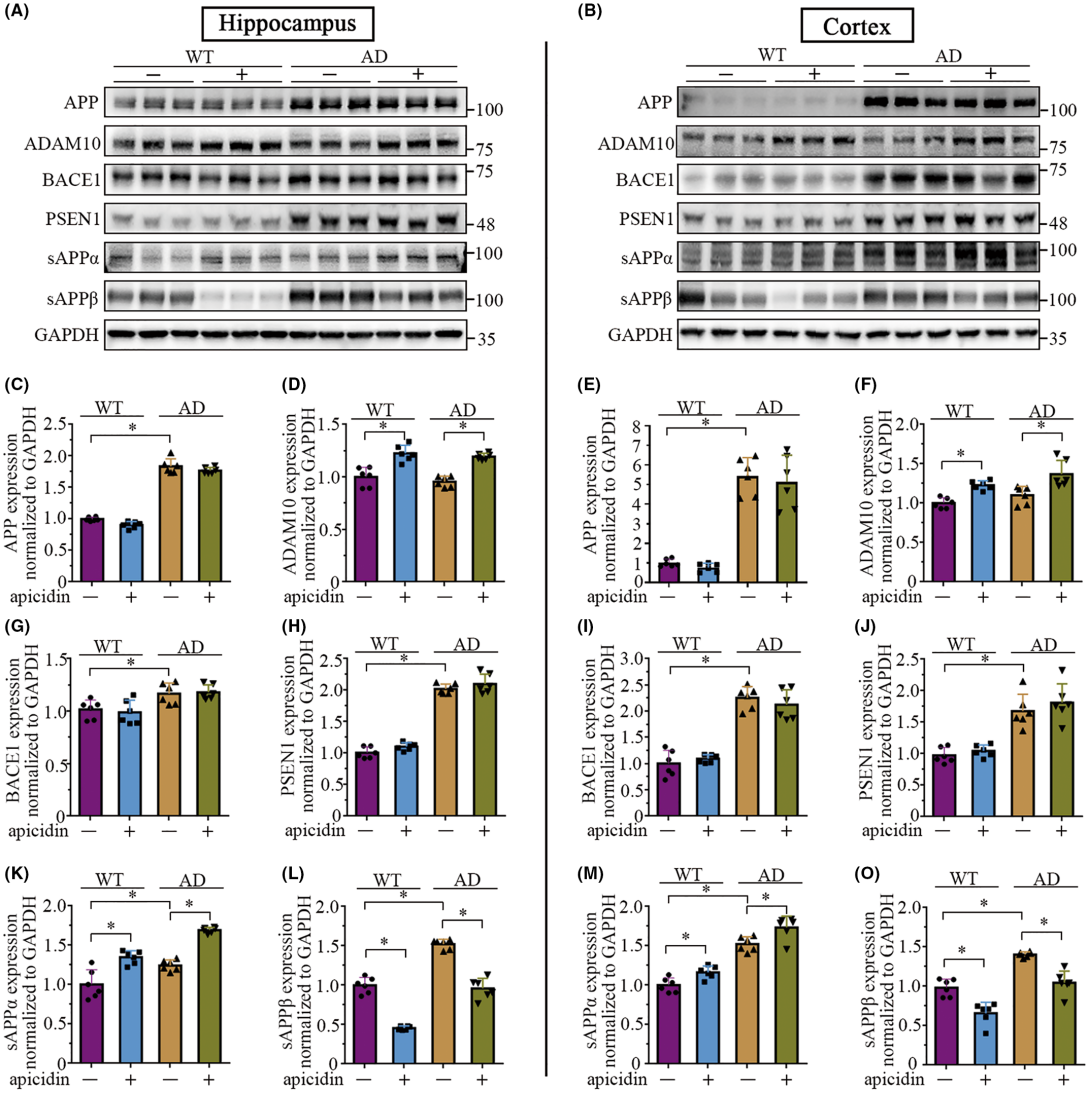
Apicidin increases the protein level of ADAM10 with an altered APP processing. (A and B) Representative Western blots of APP, ADAM10, BACE1, PSEN1, sAPPα, and sAPPβ in the hippocampus (A) and cortex (B) of WT and AD mice, in the absence or presence of apicidin, respectively. (C–O) Quantification of Western blots from the hippocampus (C–L) and cortex (E–O). Two‐way ANOVA with multiple comparisons test was used for all comparisons. The graphs represent mean ± SEM. *p < 0.05; *n* = 6.

In our previous study, we found that apicidin increased the expression of ADAM10 in cultured cells.^20^ Experimental evidence suggests that ADAM10 may exert a neuroprotective effect and may enhance learning and memory processes by affecting the nonamyloidogenic processing of APP.^24^, ^25^ To further investigate whether the reduced amyloid‐β load was associated with ADAM10 and APP processing, we assessed the effect of apicidin on the expression APP, ADAM10, BACE1, and PS1, which are also involved in the proteolytic processing of APP. Figure 5A,B shows representative Western blots demonstrating significant changes in ADAM10 and sAPPα/sAPPβ expression relative to GAPDH expression (loading control) in both the hippocampus and cortex of WT or AD mice after treatment with apicidin. In addition, the APP, BACE1, and PSEN1 levels were not altered. The quantified Western blotting results are shown in the lower panel (Figure 5C–O). Two‐way ANOVA revealed a significant effect of genotype on the APP [Hippocampus: *F*(1, 20) = 874.4, *p* < 0.0001; Cortex: *F*(1, 20) = 165.4, *p* < 0.0001], BACE1 [Hippocampus: *F*(1, 20) = 27.07, *p* < 0.0001; Cortex: *F*(1, 20) = 234.9, *p* < 0.0001], PS1 [Hippocampus: *F*(1, 20) = 707.7, *p* < 0.0001; Cortex: *F*(1, 20) = 72.38, *p* < 0.0001] and sAPPα [Hippocampus: *F*(1, 20) = 159.5, *p* < 0.0001; Cortex: *F*(1, 20) = 196.0, *p* < 0.0001]/sAPPβ [Hippocampus: *F*(1, 20) = 286.9, *p* < 0.0001; Cortex: *F*(1, 20) = 88.08, *p* < 0.0001] levels in the hippocampus and cortex. Multiple‐comparisons indicated that the ADAM10 [Hippocampus: P(WT) < 0.0001, P(AD) < 0.0001; Cortex: P(WT) = 0.0031, P(AD) = 0.0006] and sAPPα [Hippocampus: P(WT) < 0.0001, P(AD) < 0.0001; Cortex: P(WT) = 0.0152, P(AD) = 0.0021] expression levels increased with apicidin treatment, and the sAPPβ expression level decreased [Hippocampus: P(WT) < 0.0001, P(AD) < 0.0001; Cortex: P(WT) < 0.0001, P(AD) < 0.0001]. Full unedited blots are in Appendix S1. These data are consistent with the in vitro findings that the expression of ADAM10 was specifically increased by apicidin. Furthermore, increased sAPPα levels and decreased sAPPβ levels suggest a decrease in amyloidogenic APP processing.

### 3.4 Apicidin treatment does not reduce tau phosphorylation in the brains of APP/PS1 mice

**FIGURE 6:**
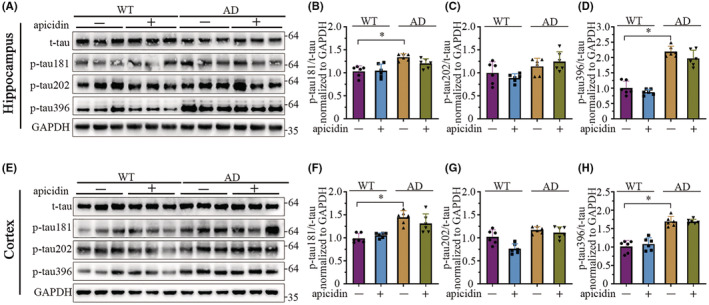
Apicidin does not decrease tau hyperphosphorylation in APP/PS1 mice. (A and E) Representative western blots showing phosphorylation‐tau proteins in the hippocampus (A) and cortex (E) of WT and AD mice. (B–D) Quantification of the hippocampus levels of phosphorylation‐tau involved in phosphorylated tau at Thr181 (B), Ser202 (C), and Ser396 (D), assayed by western blotting. (F–G) Quantification of the cortex levels of phosphorylation‐tau involved in phosphorylated tau at Thr181 (F), Ser202 (G), and Ser396 (H), assayed by western blotting. Quantified results were normalized to total Tau (t‐tau) expression. Two‐way ANOVA with multiple comparisons test was used for all comparisons. The graphs represent mean ± SEM. *p < 0.05; *n* = 6.

Excessive tau phosphorylation at Thr181, Ser202, and Ser396 has been previously shown to inhibit the physiological binding of tau to microtubules, thus impairing its physiological function in AD.^27^, ^28^, ^29^ Current studies suggest that HDAC inhibitors may also improve memory loss in AD mouse models by inhibiting tau phosphorylation.^13^, ^14^, ^15^ Next, we evaluated the effect of apicidin treatment on tau phosphorylation at disease‐relevant residues in the brains of AD mice. Lysates of the hippocampus and cortex regions of mouse brains were immunoblotted using antibodies against total tau and tau phosphorylated at different residues (Figure 6A,E). As shown in Figure 6B–H, the basal levels of phospho‐tau Thr181 [Hippocampus: *F*(1, 20) = 39.82, *p* < 0.0001; Cortex: *F*(1, 20) = 43.37, *p* < 0.0001] and Ser396 [Hippocampus: *F*(1, 20) = 285.9, *p* < 0.0001; Cortex: *F*(1, 20) = 199.3, *p* < 0.0001] were significantly higher in the AD mice than in the WT mice according to two‐way ANOVA. In contrast, phospho‐tau Thr202 [Hippocampus: *F*(1, 20) = 3.359, *p* = 0.0818; Cortex: *F*(1, 20) = 25.97, *p* = 0.0897] was not statistically significantly different. Interestingly, we did not observe a significant decrease in the levels of phospho‐tau Thr181 [Hippocampus: P(AD) = 0.0517; Cortex: P(AD) = 0.1791], Ser202 [Hippocampus: P(AD) = 0.3984; Cortex: P(AD) = 0.4635], or Ser396 [Hippocampus: P(AD) = 0.0623; Cortex: P(AD) = 0.9968] in the AD mice treated with apicidin (Figure 6B–H). Full unedited blots are in Appendix S1. These results indicated that apicidin did not reduce the phosphorylation of tau at Thr181, Ser202, and Ser396 in AD mice.

## 4 DISCUSSION

The prevalence of AD increases annually with the aging of the population, and it has become an important problem for societies worldwide. The development of an effective treatment to relieve the symptoms of AD is urgently needed. Dysregulation of the epigenetic landscape plays a critical role in the development of AD.^8^ The accumulation of histone acetylation modification is strongly associated with aging and is dysregulated in AD.^9^ In recent years, several HDAC inhibitors, such as valproic acid (VPA), sodium 4‐phenylbutyrate (4‐PBA), and nicotinamide (VB3), have been found to improve learning and memory deficits in AD mouse models.^12^, ^13^, ^15^ Our previous study showed that apicidin treatment could significantly increase the expression of ADAM10, improve the levels of the alpha‐site C‐terminal fragment (αCTF) of APP, and ultimately reduce the production of beta‐amyloid peptide 1–42 (Aβ42) in cultured cells; however, this effect was not observed in animal models of dementia. In the present study, we aimed to further investigate the neuroprotective effect of apicidin in APP/PS1 double transgenic mice, and our data showed that (i) treatment of APP/PS1 mice with apicidin for two mo reversed spatial reference memory deficit and contextual fear memory phenotypes of the mice; (ii) treatment with apicidin attenuated the formation of Aβ‐enriched plaques and decreased levels of soluble and insoluble Aβ40/42 in the brains of AD mice; and (iii) apicidin treatment significantly increased the expression of ADAM10, improved the levels of sAPPα, and reduced the production of sAPPβ in the hippocampus and cortex of APP/PS1 mice. Furthermore, apicidin did not alter the levels of phosphorylated tau. Therefore, the findings of this study suggest that apicidin has significant potential for use as a therapeutic agent to restore memory function in AD.

It is reported that apicidin has low toxicity in vivo and in vitro, which is consistent with our previous results in SH‐SY5Y cells.^19^, ^30^ Apicidin can effectively penetrate the blood–brain barrier.^31^ The pharmacokinetics of apicidin has been investigated in various species.^31^, ^32^, ^33^ According to the physiologically based pharmacokinetic (PBPK) model, the peak concentration of apicidin in the brain of mice is roughly 189 ng/g after intraperitoneal injection of apicidin at 3.5 mg/kg.^31^ However, the oral bioavailability of apicidin was found to be 19.3% and 14.2% in fasting and nonfasting rats, respectively.^31^, ^32^ The reason for the low oral availability of apicidin was revealed in another study, namely intestinal absorption is the rate‐determining step for oral absorption of apicidin.^33^ In our study, we administered the drug via intraperitoneal injection, which is a suitable route for chronic treatments, minimizes the stress experienced by laboratory rodents,^34^ and importantly avoids the low absorption efficiency of apicidin in the intestine.

APPswe/PSEN1dE9 (APP/PS1) mice are widely used in AD research; these mice begin to develop Aβ deposits in the hippocampus and cortex by 6 months of age and exhibit progressive memory deficits.^35^, ^36^ In our study, the MWM test and contextual fear conditioning test, which are widely used in memory research, were conducted to evaluate spatial reference memory and contextual fear memory in the mice, respectively (Figure 2A–H). First, the APP/PS1 mice showed worse performance in behavioral tests than the WT mice, which is consistent with our previous reports.^37^ Visual ability must be accounted for when conducting the MWM test with mice because differences in the performance of many tasks may occur due to visual deficits rather than differences in higher‐order cognitive functions.^38^ In the visible platform test, we found no significant difference in motor or visual discrimination in mice after treatment with apicidin or normal saline (Figure 2A,B). This result suggested that apicidin treatment did not influence the visual and motor abilities of the mice. Interestingly, APP/PS1 mice showed significantly slower swimming rates than WT mice in the visible platform test based on the calculation of the latency and path length to the visible platform (Figure S1C). In line with this, APP/PS1 mice have an increased tendency to float during the course of swimming.^39^ Considering the strong correlation between floating and swim speed, the difference in floating may be the main reason for the slow swimming speed of the APP/PS1 mice.

In our study, the WT mice started to exhibit predominantly shorter latency early during the hidden platform training trial, which occurred in the APP/PS1 mice only after extensive training (Figure 2C). Latency reflects differences in spatial memory performance that are closely related to hippocampal function.^40^ Our data showed that treatment with apicidin markedly ameliorated latency in the APP/PS1 mice, especially during days 5–8 in the hidden platform training trial. Importantly, amelioration of learning and memory deficits was manifested during the probe trial. The WT mice and APP/PS1 mice treated with apicidin developed a specific preference for the target quadrant, whereas the APP/PS1 mice treated with ns used less efficient search strategies (Figure 2D). In addition, this difference was also reflected in the number of times that the target area was crossed (Figure 2E) and time spent in the target quadrant (Figure 2F). It has been reported that the contextual fear memory of APP/PS1 mice is impaired as early as 6 months of age, as shown by freezing behavior during fear conditioning tests.^12^ As expected, in the contextual fear conditioning test, APP/PS1 mice treated with apicidin exhibited decreased freezing compared to APP/PS1 mice treated with ns (Figure 2G). These results suggested that apicidin treatment attenuates spatial and contextual memory deficits in APP/PS1 mice.

Previous studies have shown that increased histone acetylation by HDAC inhibitors mainly induced the sprouting of dendrites and increased the number of synapses, ultimately enhancing long‐term memories.^41^, ^42^ Moreover, HDAC inhibition or silencing improved memory performance in mouse models of dementia.^8^, ^43^ Although potential memory enhancement by HDAC inhibitors may contribute to behavioral improvement, it is clear that the Aβ and hyperphosphorylated tau levels correlate more strongly with memory impairment in APP/PS1 mice.^44^ In our study, APP/PS1 mice exhibited many more 6E10‐positive plaques either in the hippocampus or in the cortex compared to the WT mice (Figure S2A,B). Interestingly, different levels of Aβ deposition were observed by 6E10 antibody and thioflavin S staining, possibly because of the different sensitivities of the methods^45^ (Figure 3A,C). Despite these differences, it is clear that both the 6E10 antibody and thioflavin S staining results suggest that apicidin treatment significantly attenuated the formation of amyloid plaques in the brains of AD mice. Although Aβ (1–40/1–42) is the main component of amyloid plaques, the solubility of Aβ (1–40/1–42) is a critical determinant of amyloidosis.^46^ One study found that the levels of soluble Aβ (1–40/1–42) are highly related to cognitive deficits in AD mice.^47^ Our study showed that apicidin treatment decreased the levels of soluble Aβ (1–40/1–42) in the hippocampus (Figure 4A) and cortex (Figure 4B) of the APP/PS1 mice, which was consistent with the results of the behavioral experiments. Additionally, we also found that insoluble Aβ (1–40/1–42) levels were reduced in the hippocampus (Figure 4A) and cortex (Figure 4B) of the APP/PS1 mice treated with apicidin, confirming the immunohistochemical findings. These results indicate that the Aβ burden in APP/PS1 mouse brains was inhibited by treatment with apicidin.

Apicidin has been used as an inhibitor of HDAC2/3 in several studies.^48^, ^49^ It is reported that A pan‐HDAC inhibitor valproic acid reduced Aβ through the γ‐secretase cleavage of APP^50^; and RGFP‐966, an inhibitor of HDAC3 decreased Aβ accumulation, whereas the potential mechanism was not elucidated.^14^ We have previously reported that apicidin promoted ADAM10 transcription through transcription factor upstream transcription factor 1 (USF1) in HDAC2/3‐dependent manner,^20^ suggesting that apicidin increases ADAM10 expression through HDAC2/3. In APP/PS1 transgenic mice, mutant PSEN1dE9 promotes the activation of γ‐secretase and enhances the cleavage of sAPPα/β, resulting in higher levels of Aβ.^35^ In line with this, we observed overexpression of APP and PS1 with increased sAPPα/β levels in the APP/PS1 mice (Figure 5A–O). Importantly, as expected, we observed that apicidin treatment increased ADAM10 expression in both the hippocampus (Figure 5D) and cortex of mice (Figure 5F). We particularly noted that the levels of sAPPβ were further decreased when sAPPα production was increased in the mice treated with apicidin, but we did not observe any changes in the BACE1 levels (Figure 5G,I,L,O). Previous studies also confirm that the enhanced nonamyloidogenic pathway of APP with increased sAPPα production reduces APP proteolysis in the amyloidogenic pathway, resulting in decreased levels of sAPPβ.^24^ Therefore, we speculate that the differentially regulated levels of sAPPα and sAPPβ by apicidin was through the overexpression of ADAM10.

Numerous studies have observed that the phosphorylation of some sites of tau is significantly increased in APP/PS1 mice despite the lack of mature tangles.^51^, ^52^, ^53^ Consistent with previous studies, we found that the level of tau phosphorylation at Thr181 and Ser396 was increased in the hippocampus (Figure 6B,D) and cortex (Figure 6F,H) of the APP/PS1 mice, but similar results were not observed for tau phosphorylation at Ser202 (Figure 6C,G). The role of HDAC2/3 in tau pathway is not well‐defined. HDAC2/3 belongs to class I HDAC family.^54^ It is reported that tubacin, a selective inhibitor of HDAC6 (class II) attenuates tau phosphorylation at selective sites.^55^ Sirtuin inhibition and phenylbutyrate, which are associated with class II/III HDAC activity, inhibit tau phosphorylation.^13^, ^15^ In the 3xTg‐AD mouse model, the HDAC3 inhibitor RGFP‐966 reduces Aβ deposition as well as the phosphorylated tau,^14^ which might be a secondary effect caused by Aβ.^54^ In our study, apicidin fails to regulate tau, which might be attributed to the different class‐specific HDAC inhibitors and the animal models used. Interestingly, one study indicated that the burden of neurofibrillary tangles involving the hyperphosphorylation of tau is increased in APP/PS1 × rTg4510 (tau P301L) mice compared to rTg4510 (tau P301L) mice only at earlier disease stages.^56^ Another study also suggested that Aβ reduction could slow the rate of tangle formation in early, but not later, phases of AD.^57^ Therefore, we consider that the reduction in the Aβ levels due to apicidin treatment (in mice aged 9–12 months) cannot reduce the level of phosphorylated tau in older APP/PS1 mice because tau phosphorylation probably occurs in an Aβ‐independent manner in later phases of AD. On the other hand, decreasing tau pathology by inhibiting HADC may require model mice with complete tau pathology rather than APP/PS1 mice.

Altogether, the present study provides evidence that apicidin treatment ameliorated memory impairment in APP/PS1 mice, and this effect was attributed to decreasing the Aβ burden by promoting the expression of ADAM10. Thus, we propose that apicidin may be a promising therapeutic strategy for attenuating AD progression.

## AUTHOR CONTRIBUTIONS

Biao Luo and Jian Chen were responsible for the experimental operation, data statistics, and manuscript drafting of the study. Gui‐Feng Zhou, Jing Tang, Qi‐Xin Wen, Xiao‐Yong Xie, Li Song, Shi‐Qi Xie, and Yan Long provided all reagents, materials, and animals. Xiao‐Tong Hu and Guo‐Jun Chen conceived the initial idea, designed the study, and participated in the manuscript revision. All authors read and approved the final manuscript.

## FUNDING INFORMATION

This work was supported by Natural Science Foundation of Chongqing (cstc2019jcyj‐msxmX0650) to X‐T Hu and Chongqing Education commission (KJZD‐K201900404) and NSFC (81971030) to G‐J Chen.

## CONFLICT OF INTEREST STATEMENT

All authors disclosed no relevant relationships.

## Supporting information


Figure S1.

Figure S2.

Appendix S1.

## Data Availability

The original contributions presented in the study are included in the article/supplementary material, further inquiries can be directed to the corresponding author.
