# Supplementary figures and images for: Apicidin attenuates memory deficits by reducing the Aβ load in APP/PS1 mice
Source: CNS Neurosci Ther. 2023 Jan 27;29(5):1300–11. doi: 10.1111/cns.14102 (PMC10068467; doi:10.1111/cns.14102)

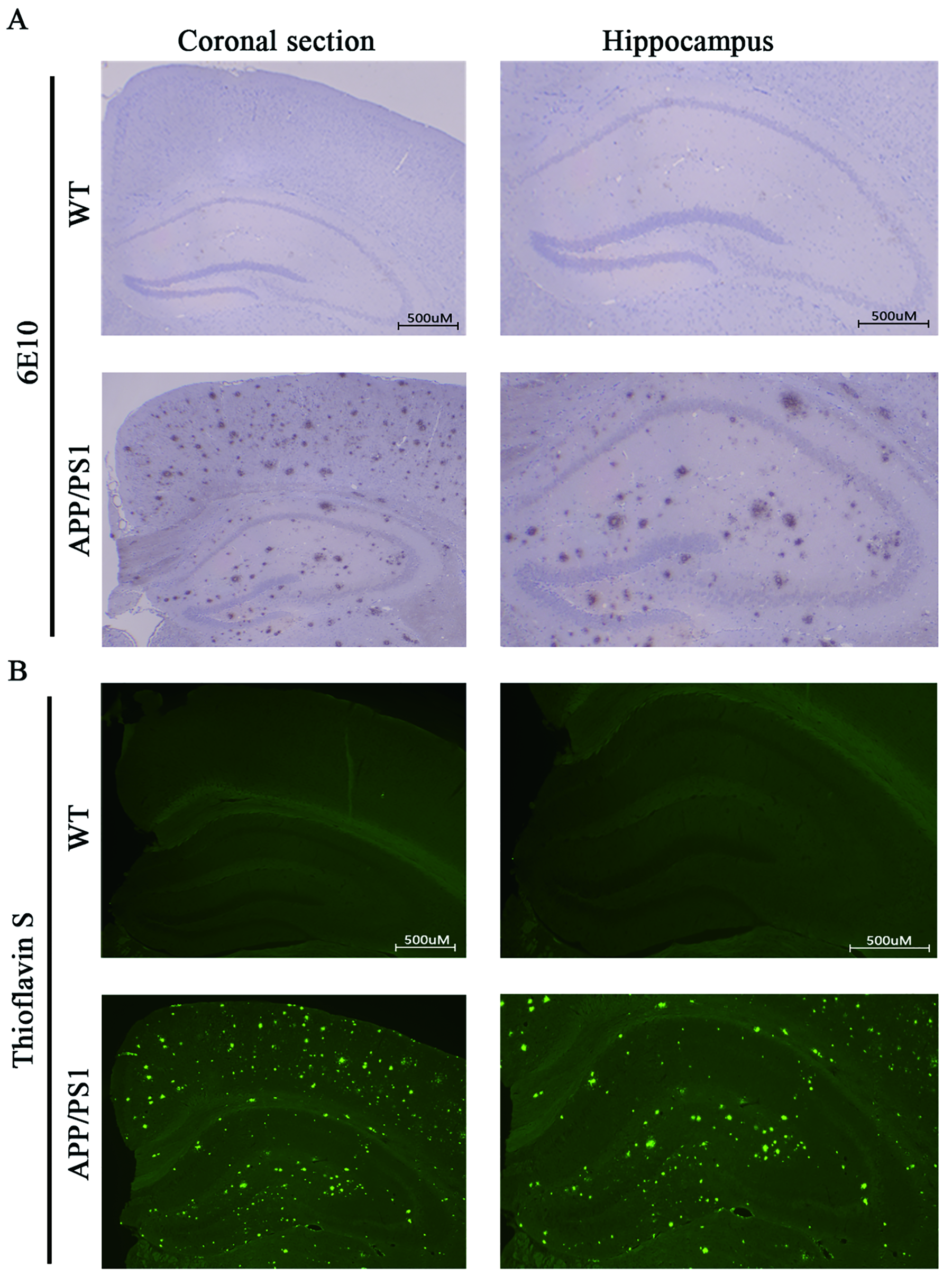

Supplement: Supplementary file 2 — Figure S2. [file CNS-29-1300-s002.tif]

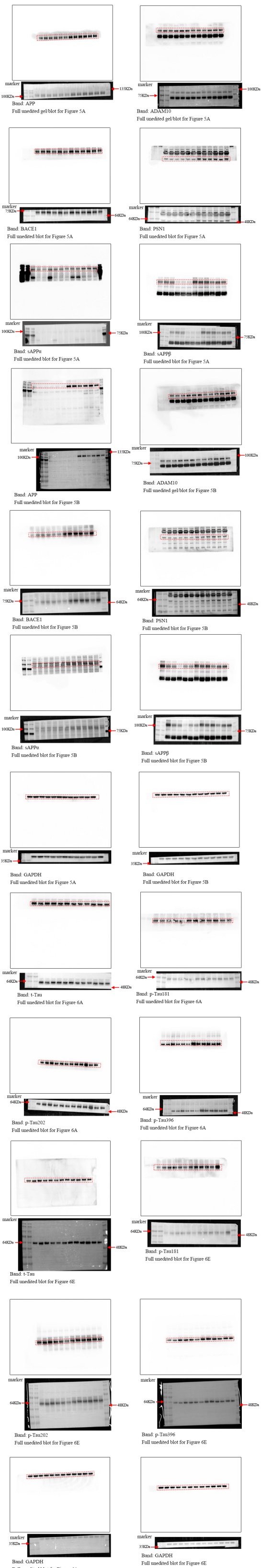

Supplement: Supplementary file 3 — Appendix S1. [file CNS-29-1300-s001.pdf]
